# Effect of Sleep Quality on the Prevalence of Sarcopenia in Older Adults: A Systematic Review with Meta-Analysis

**DOI:** 10.3390/jcm8122156

**Published:** 2019-12-06

**Authors:** Jacobo Á. Rubio-Arias, Raquel Rodríguez-Fernández, Luis Andreu, Luis M. Martínez-Aranda, Alejandro Martínez-Rodriguez, Domingo J. Ramos-Campo

**Affiliations:** 1 LFE Research Group, Department of Health and Human Performance, Faculty of Physical Activity and Sport Science-INEF, Universidad Politécnica de Madrid, 28040 Madrid, Spain; 2 Department of Methodology of Behavioral Sciences, Faculty of Psychology, Universidad Nacional de Educación a Distancia (UNED), 28040 Madrid, Spain; rrodriguez@psi.uned.es; 3 International Chair of Sports Medicine, Universidad Católica San Antonio de Murcia (UCAM), 30107 Murcia, Spain; landreu@ucam.edu; 4 Faculty of Sports, Universidad Católica San Antonio de Murcia (UCAM), 30107 Murcia, Spain; lmmartinez2@ucam.edu (L.M.M.-A.); Djramos@ucam.edu (D.J.R.-C.); 5 Neuroscience of Human Movement Research Group (Neuromove), Universidad Católica San Antonio de Murcia (UCAM), 30107 Murcia, Spain; 6 Department of Analytical Chemistry, Nutrition and Food Science, Faculty of Science, Alicante University, 03690 Alicante, Spain

**Keywords:** muscle-mass, sleep efficiency, sleep duration, insomnia

## Abstract

Sarcopenia is an age-related condition. However, the prevalence of sarcopenia may increase due to a range of other factors, such as sleep quality/duration. Therefore, the aim of the study is to conduct a systematic review with meta-analysis to determine the prevalence of sarcopenia in older adults based on their self-reported sleep duration. Methods: Three electronic databases were used—PubMed-Medline, Web of Science, and Cochrane Library. We included studies that measured the prevalence of sarcopenia, divided according to sleep quality and excluded studies (a) involving populations with neuromuscular pathologies, (b) not showing prevalence values (cases/control) on sarcopenia, and (c) not including classificatory models to determine sleep quality. Results: high prevalence values in older adults with both long and short sleep duration were shown. However, prevalence values were higher in those with inadequate sleep (<6–8 h or low efficiency) (OR 0.76; 95% CI (0.70–0.83); Q = 1.446; *p* = 0.695; test for overall effect, Z = 6.01, *p* < 0.00001). Likewise, higher prevalence levels were shown in men (OR 1.61; 95% CI (0.82–3.16); Q = 11.80; *p* = 0.0189) compared to women (OR 0.77; 95% CI (0.29–2.03); Q = 21.35; *p* = 0.0003). Therefore, the prevalence of sarcopenia appears to be associated with sleep quality, with higher prevalence values in older adults who have inadequate sleep.

## 1. Introduction

Together with the increment of the world population and life span over the years, a parallel increase in chronic diseases [1] has been observed, such as sarcopenia. This pathology has become a serious global public health problem [2] since it can lead to a considerable increase in costs due to the frequency and duration of hospitalization, as well as an increase in the number of falls as a consequence of muscle weakness [3,4]. In addition, people who suffer a high loss of muscle mass have an increased risk of other health problems, such as heart failure, chronic obstructive pulmonary diseases, kidney failure [5] or osteoporosis [6] and, therefore, a greater risk of bone fracture, turning sarcopenia into a major health problem that should be addressed in order to determine the possible factors associated with sarcopenia.

Sarcopenia has been defined as a decrease and deterioration of muscle mass associated with aging [7]. Thus, the skeletal muscle mass is progressively lost during aging and is partially replaced by fat and connective tissue due to a reduction and leakage of type II fibers generated by a slow degenerative neurological process [8]. This decrease in muscle mass due to aging also generates a decrease in muscle strength and, therefore, a physical disability generating a functional limitation (activities of the daily life) as well as a decrease in the life quality [9,10] with an associated increase in the risk of mortality [11,12]. In addition, this muscle mass loss has a greater impact on women during menopause as a consequence of the decrease in the estrogen levels after the fifth decade of life [13]. Sex differences in body composition are well known [14], with men having a higher cross-sectional area in skeletal muscle than women and greater muscle in the upper body [15]. Additionally, women are at higher risk of developing sarcopenic obesity due to increased fat and lower muscle mass [14]. Nevertheless, the results of prevalence related to sex are inconsistent [2]. In these circumstances, efforts are required to identify the factors associated with sarcopenia and to implement interventions for the prevention or the incidence reduction of this pathology among the elderly population [16], considering sex as a modifying variable.

However, the loss of muscle mass (sarcopenia) is not only related to age and sex but also depends on a number of endogenous and exogenous factors that influence the prevalence values of sarcopenia. The most studied and validated factors that can generate an effect on the sarcopenia are age (main moderating variable), genetic factors, birth weight, early growth, diet, physical activity, other chronic diseases, and hormonal changes (secondary variables) [17,18]. In line with this, a recent systematic review with meta-analysis on the general population [2] concludes that the prevalence of sarcopenia can be modified by other factors such as race, nutrition, quality of life, and sex among others.

Nonetheless, the scientific literature shows a gap between the role that sleep quality could play and the effects on the prevalence of sarcopenia. As Buchmann et al. (2016) [19] suggest, sleep is associated with a biological and mental regeneration process. Moreover, Vitale et al. (2019) [20] reported that the maintenance of circadian rhythms can be altered by aging and the development of many chronic diseases, including sarcopenia. The preservation of circadian rhythm is very important for the sustainment of cellular physiology, metabolism, and function in the skeletal muscle. Therefore, people who have an inadequate sleeping time could have an increased risk of mortality compared to those who sleep the recommended daily hours [21]. In addition, under low sleep conditions, the cognitive abilities might be affected and can be an increment in the risk of mortality and falling in older adults [22]. In this way, sex may also play a significant role in sleep quality, due to the fact that women have a greater predisposition of insomnia found among different criteria, frequencies, and duration [23]. Nevertheless, the association between muscle mass, sleep quality, and sex is not clear yet and no studies have been found to support such affirmation.

Certainly, the lack of sleep not only leads to a deterioration of cognitive abilities but can also have a negative effect at the cellular level on muscle physiology. It impairs muscle recovery due to increased stimulation of protein degradation, which is detrimental for protein synthesis and promotes muscle atrophy [24]. In addition to the negative effect on muscle mass, it has been associated with cardiovascular disease [25], type II diabetes [26], hypertension [25], obesity [27], and colorectal cancer [28]. In this regard, public health should include sleep duration/quality as one of the risk factors associated with a large number of diseases.

Some correlational studies have determined the effect of sleep duration on muscle mass, showing that less sleep duration or quality leads to a loss of muscle mass [29]. However, no meta-analyses addressing the effect of sleep duration or quality on the prevalence of sarcopenia have been found. Therefore, the objectives of this systematic review with meta-analysis are (1) to analyze the overall prevalence of sarcopenia in people with optimal sleep duration/quality compared to those with inadequate sleep quality, (2) to analyze whether the prevalence of sarcopenia is correlated to the sex of the participants. Our starting hypothesis is that people with poor rest show a higher prevalence of sarcopenia than those who rest in better conditions and, in addition, men will have a lower prevalence compared to women.

## 2. Experimental Section

### 2.1. Study Design

A systematic review with meta-analysis was performed following the recommendations of PRISMA (preferred reporting items for systematic review and meta-analysis) [30]. All the analyses were performed in duplicate (J.A.R.A. and L.A.), all disagreements on inclusion/exclusion were discussed and resolved by consensus. The extrinsic characteristics of the publications and the substantive characteristics—population, sex, associated pathology, habits of alcohol, tobacco, physical activity, age, and BMI—were extracted from the studies that were finally included in the quantitative analysis. Finally, the methodological characteristics—duration of sleep, quality of sleep, muscular mass and presence or not of sarcopenia—were also considered. All subjects included in the analysis were classified as cases or control differentiating sleep and sex.

### 2.2. Search and Data Sources

Three electronic databases were used: PubMed-Medline, Web of Science, and Cochrane Library. The search was conducted without search date restriction and ended on 28 July 2019. The key search words and strategy were “Sleep Disorders” OR “Sleep Deprivation” OR “Sleep Hygiene” OR “Sleep duration” OR insomnia OR sleep* and “muscle mass” OR “muscular atrophy” OR sarcopenia.

### 2.3. Data extraction and Inclusion/Exclusion Criteria

The following inclusion criteria were considered: prevalence studies analyzing the effect of sleep on sarcopenia and conducted in adults (>40). Studies were excluded if they included (a) populations with neuromuscular pathologies, (b) studies that did not show prevalence values (cases/control) on sarcopenia, and (c) studies that did not include classificatory models that allowed sleep quality to be determined.

### 2.4. Outcomes

The variables to determine the prevalence of sarcopenia as a function of sleep were (1) the presence or absence of sarcopenia, and (2) sleep quality. Sleep can be assessed to estimate adequate or inadequate sleep in terms of quality or duration in different ways. For the questionnaires, adequate-sleep (sleep well) for those who obtained between very good or quite good in the percentage of quality and not-adequate-sleep (sleep bad), rather bad or very bad were considered [19]. Regarding the hours of sleep, they were considered inadequate (<6–8) and adequate (≥8), following the recommendations of the National Sleep Foundation [21].

### 2.5. Assessment of Risk of Bias

The Q-index was used to assess the methodological quality, a scale that allows us to quantify the bias, obtaining a final score between 0 (minimum quality) and 1 (maximum quality). This rescaled quality range (called Qi in MetaXL) has a monotonic relationship to ICC bias, defined as the variance of the study bias divided by the sum of the variance of bias within and between studies [31,32]. Quality analysis was performed on each study based on the method of assessing sleep quality and sarcopenia, giving higher preference to the studies that measured the sleep with instruments previously validated for this purpose, as well as sarcopenia with DXA or BIA. Therefore, the studies using both DXA and a validated questionnaire to analyze sleep quality were scored with 1. The following criteria were conducted:

(Q1) Were the target population and the observation period well defined?: yes = 1 and no = 0;

(Q2) Diagnostic criteria, use of diagnostic system reported: sarcopenia = DXA or BIA and sleep quality = instruments validated = 1 and own system/symptoms described/no system/not specified = 0;

(Q3) Method of case ascertainment: community survey/multiple institutions = 2, inpatient/inpatients and outpatients/case registers = 1, and not specified = 0;

(Q4) Administration of measurement protocol: administered interview = 3, systematic case-note review = 2, chart diagnosis/case records = 1 and not specified = 0;

(Q5) Catchment area: broadly representative (national or multi-site survey) = 2, small area/not representative (single community, single university) = 1, and convenience sampling/other (primary care sample/treatment group) = 0; and

(Q6) Prevalence measure: point prevalence (e.g., one month) = 2, 12-month prevalence = 1 and lifetime prevalence = 0.

In addition, the overall publication bias of the studies was analyzed using the funnel plot, dividing between older adults who slept well and those who had inadequate sleep.

### 2.6. Data Synthesis and Statistical Analysis

Meta-analysis and statistical analysis were performed using MetaXL software version 2.0 (Sunrise Beach, Queensland, Australia). The prevalence of sarcopenia (cases vs. control) was initially calculated in the included studies for random-effects model analysis (no transformation methods) and then recalculated under a rescaled quality of bias effects model. For the analysis, the sleep category was considered (sleep well and sleep poorly) for the calculation of the overall prevalence of sarcopenia, and this method was applied under the random-effects model and effects in quality of the rescaled bias using three possible transformations (None, Logit, and Arcsine) [31,32] to contrast the effects of the prevalence of sarcopenia. In all cases, pooled prevalence values were shown, 95% CI, heterogeneity *I*^2^, Cochran’s Q, chi^2^, *p*, tau^2^. On the other hand, the grouped odds ratios (OR) and their IC95% were also calculated following a model of “quality effects” [31,33] to analyze the association between those who sleep well (control) and those who sleep poorly (effect). In addition, OR and sleep category analysis were estimated, excluding studies that did not report participants’ total hours of sleep and only showed sleep quality [19,34]. Heterogeneity between studies was conducted using the *I*^2^ statistic, and the variation between studies was calculated using the tau^2^ statistic (τ^2^) [35]. *I*^2^ values between 30–60% were considered as moderate levels of heterogeneity, while a value of τ^2^ > 1 suggested the presence of substantial statistical heterogeneity. The minimum level of significance was set as *p* ≤ 0.05.

## 3. Results

### 3.1. General Characteristics of the Studies

**Figure 1:**
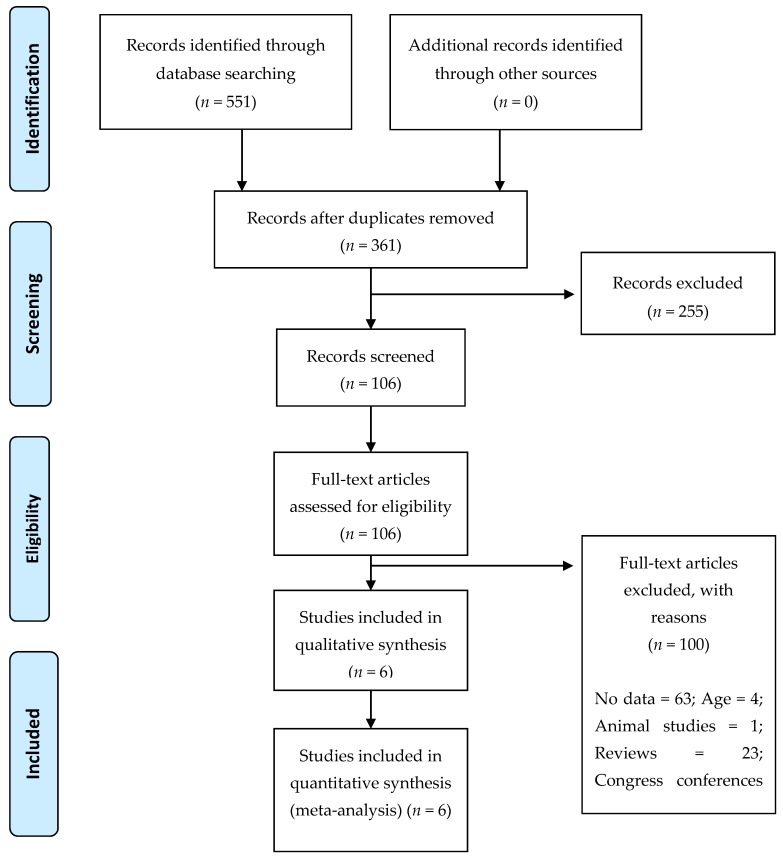
Flow diagram of the process of study selection.

A total of 551 items were identified from the selected databases, and 0/0 items were included from other sources. After the removal of duplicated articles from the different databases, 361 titles and abstracts, as well as 106 articles, were reviewed, and 255 were removed. Finally, statistical analysis was performed on a total of 6 studies [19,34,36,37,38,39] (5 were performed in Asia and only 1 in Europe; Figure 1), with a mean age of 68.7 years (range = 44–80 years). Table 1 shows the descriptive characteristics of the studies included in our analysis. The selected studies included 6405 (990 cases and 5415 controls) older adults with adequate sleep and 12,708 (1762 cases and 10,946 controls) adults with inadequate sleep. However, only four studies contained data divided by sex (1232 men) including 142 cases with adequate sleep and 95 cases with inadequate sleep. In addition, 1381 women were enrolled in the four studies, with 109 cases in the adequate sleep group and 118 cases in the inadequate sleep group.

### 3.2. Quality of the Studies

The assessment of the methodological quality of the studies included in the quantitative analysis is summarised in Table 2.

**Figure 2:**
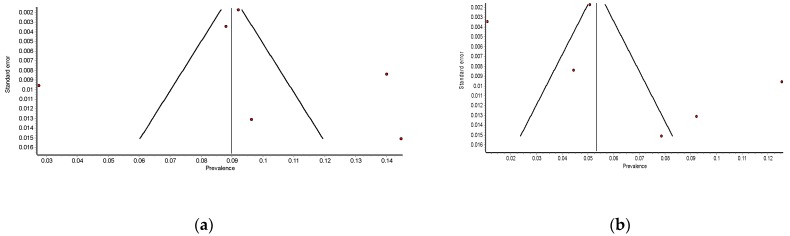
Funnel plot of the meta-analysis of the published studies. Each plotted point represents the.standard error (SE) and the prevalence. (**a**) Sleep well, (**b**) sleep poorly.

When estimating a study quality, the mean score was 0.833 (range: 0.7–1). Three studies used bioelectrical impedance analysis (BIA) [33,36], three DXA [19,38,39] and only one study used a self-administered questionnaire [34]. Furthermore, three studies used the Pittsburg Sleep Quality Index (PSQI) [19,34,36], and the other three assessed the sleep duration/quality with self-reports. In addition, two studies [34,37] did not specify the case measurement system. The funnel plots suggest the presence of significant publication bias (Figure 2).

### 3.3. Meta-Analysis

**Figure 3:**
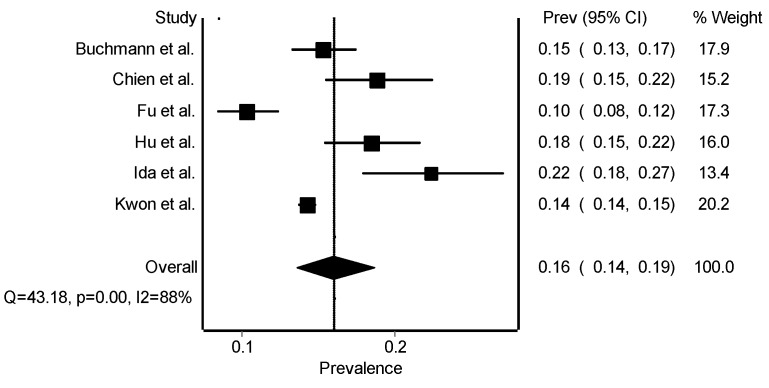
Overall prevalence of studies included in the analysis (method: random effects).

The overall results of the prevalence of sarcopenia in the studies included in our meta-analysis revealed a high prevalence (Figure 3). When the methodological quality of the studies was considered in the results (Table 2), the prevalence was decreased (Total = 19,677 participants, 2858 cases; 0.144, 95% CI (0.100–0.189); Q = 41.90, *p* = 0.0000) but maintaining a high heterogeneity (*I*^2^ = 88).

#### Results by Sleep Categories

The effects of the prevalence of sarcopenia sorted by categories are shown in Table 3. The prevalence values are grouped by sleep quality categories (sleep well and sleep poorly).

People who sleep well had lower values than those who sleep poorly and the prevalence in all the sub-analyses was high, suggesting a prevalence of sarcopenia independently of the category. However, the OR value was not significant (OR 0.81; 95% CI (0.41–1.60); Q = 34.04; *p* = 0.0000; test for overall effect, Z = 0.12, *p* = 0.91) when analysing the relationship between sarcopenia and sleep quality. Nonetheless, when the relationship between sleep quality and sarcopenia was analyzed after excluding the Buchmann et al. [19] and Ida et al. [34] studies from the analysis due to high heterogeneity, the sleep quality was associated with sarcopenia (OR 0.76; 95% CI (0.70–0.83); Q = 1.446; *p* = 0.695; test for overall effect, Z = 6.01, *p* < 0.00001). Likewise, the subjects who self-reported fewer sleeping hours showed a higher prevalence of sarcopenia.

**Figure 4:**
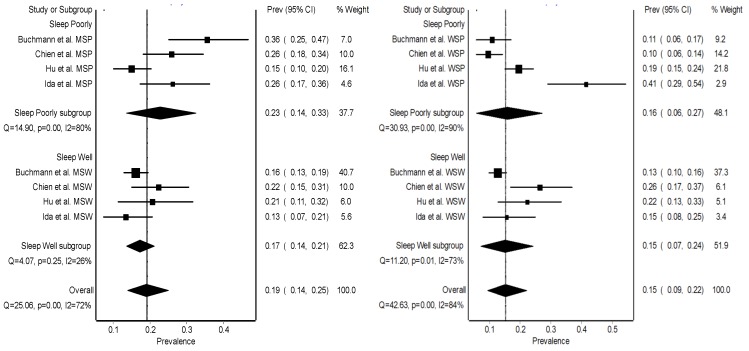
Prevalence of sarcopenia according to the sex of the participants. MSP, men sleep poorly; MSW, men sleep well; SWP, women sleep poorly; and WSW, women sleep well.

Due to the high heterogeneity of the studies included in the meta-analysis, a gender analysis of prevalence was performed (Figure 4). Only four studies provided sex-dependent data. Non-significant associations for men (OR 1.61; 95% CI (0.82–3.16); Q = 11.80; *p* = 0.0189) or women (OR 0.77; 95% CI (0.29–2.03); Q = 21.35; *p* = 0.0003) were observed. However, the heterogeneity still showed high value in all the sub-analyses that were performed (including the quality of the studies and without any transformation), due to the heterogeneity of the methodologies, the types of studies and other parameters that were not taken into consideration for the analysis of the prevalence of sarcopenia.

## 4. Discussion

The main finding of this research is that those subjects having inadequate sleep show a higher prevalence of sarcopenia values than those who reported adequate sleep. In addition, our results revealed a high prevalence of sarcopenia in older adults.

The results showed that a higher prevalence of sarcopenia values from those who do not sleep adequately were almost twice the value of the grouped prevalence, according to the model and the transformation that were used in the analysis. In line with our findings, Chien et al. [36], observed a significant association between sleep duration and the prevalence of sarcopenia on a sample of 488 adults (224 men and 264 women) from Taiwan, even though the assessment of sarcopenia was performed by electrical bioimpedance (BIA). Moreover, in the only study carried out in Europe, focused on the German subjects [19], similar results to those described above were observed, with the addition of the association between the sleep length and the quantity of muscle mass and recommending longitudinal studies to better understand the potential association. Similarly, Hu et al. [38] observed a relationship between sleep hours and sarcopenia in a Chinese cohort (*n* = 920, 95 cases). However, in this case, a U-shaped association in the prevalence of sarcopenia was obtained, in which older adults with short or long sleep length obtained higher values compared to those with normal sleep duration.

One plausible explanation to this findings is that the participants with an inefficient sleep may have differences in hormonal regulation (anabolic and catabolic balance), with elevated levels of cortisol (catabolic hormone promoting protein degradation), and low levels of IGF-1 (anabolic hormones promoting protein synthesis), developing a positive balance towards muscle degradation and, therefore, favoring the loss of muscle mass [19,40]. Likewise, Buchmann et al. [19] also observed elevated c-reactive protein (CRP) values. CRP is a pro-inflammatory cytokine and has been proposed as a possible cause of muscular atrophy [41] and also associated with sleep deprivation in high concentrations [42]. The sleep restriction generates hormonal imbalances and pro-inflammatory effects, favoring the loss of muscle mass with age. This could be one reason for the higher prevalence of sarcopenia values of sarcopenia in people with inadequate sleep. We must consider that the losses of muscle strength and muscle mass are associated and, therefore, related to a decrease in the functional capacity and quality of life [43]. Further studies to determine the effects of sleep deprivation in patients diagnosed with sarcopenia are necessary.

Interestingly, our results suggest a higher prevalence of sarcopenia in men compared to women (men = 0.19, 95% CI (0.14–0.25); women = 0.15, 95% CI (0.09–0.22)). These results are in line with previous studies in which the prevalence of sarcopenia can occur at earlier ages, as shown by Kwon et al. [39]. They observed a prevalence of sarcopenia of 14.3% in a group of 16,148 Koreans (44.1 ± 0.2 years), being higher in men (18.7%) than in women (9.7%). This can be explained by the fact that men had a higher muscle mass compared to women, but also a larger magnitude in muscle decrease was observed in men versus women as age increased [14]. On the contrary, in previous studies where the prevalence of sarcopenia was identified at different age intervals, a lower prevalence in men compared to women was observed [44]. Although age is the main causal effect of sarcopenia, the prediction ratio of increased sarcopenia based on age is difficult to verify, due to the multitude of factors that could have an influence in the prevalence values [45]. Nonetheless, it is estimated that the prevalence of clinically significant sarcopenia ranges from 8.8% in elderly women to 17.5% in elderly men, but it should be noted that these values may be higher or lower depending on the environmental factors [46]. In our study, only four articles considered gender as a sarcopenia-modifying variable, with very different methodologies and difficult interpretation. Therefore, the effect of sex on the prevalence of sarcopenia is unclear and more studies are needed to determine this interaction.

In summary, a direct association between sleep duration and prevalence of sarcopenia were confirmed in all the studies included in the quantitative data analysis. However, the interaction of gender and sleep duration/quality is not entirely clear. Hu et al. [38] observed that the prevalence of sarcopenia due to sleep deprivation was more pronounced in women. Similar results were described by Chien et al. [36] and Ida et al. [34]. However, Buchmann et al. [19] reported poor associations between sleep deprivation and the prevalence of sarcopenia in women. This discrepancy could be justified based on the ethnicity of the participants [2] or the age range difference between the studies. This higher and more evident prevalence in women could be associated with the negative effects of menopause. Thus, a decrease in the estrogen levels during menopause could play a potential role in decreasing the muscle mass after the fifth decade of life [13]. In addition, muscle mass seems to play an important role in osteoporosis in women, since muscle contractions involve a mechanical load on the bone that could promote the rate of bone regeneration [47]. Therefore, it could be stated that there is a close link between muscle strength, muscle mass, and bone tissue [48]; and that menopausal women are a sensitive population for the prevalence of sarcopenia, although to determine the gender role of this prevalence more studies would be needed. In addition, physical activity and programmed exercise should be considered, as it could play a relevant role in the prevalence of sarcopenia by improving sleep quality [49].

Finally, the results of this review should be interpreted with caution, since several limitations could be influencing them. For example, the high heterogeneity shown in the analyses could not be corrected by means of the rescaled bias scale. Another point to consider is the origin of the studied population. In five of the analyzed studies, the subjects cohort was from Asia, while only one single study was performed using a European population and the way of measuring the cut-off point and the provenance could be biasing the results [2], resulting in different tendencies between men and women. Other limitations are the way in which the sarcopenia [6] is conceptualized, resulting in prevalence variations due to the different techniques developed to measure sarcopenia, as well as the classification of the pathologic incidence [50] and that sleep quality was only self-reported throughout questionnaires and not objectively monitored; and the low number of articles included for the data for quantitative analysis (low security measure).

## 5. Conclusions

The main conclusion is the observed association between sleep duration/quality and the prevalence of sarcopenia. In addition, this prevalence seems to be higher in men than in women. These results could have a practical application for the public health since it can help us to consider sleep quality as a risk factor, as well as the need to incorporate therapies in order to improve the sleep quality and to reduce the negative effects of age-associated sarcopenia.

## Figures and Tables

**Table 1:** Characteristics of the included studies in the meta-analysis.

Extrinsic Variables	Substantive Characteristics											Methodological Characteristics								
															Sleep Well			Sleep Poorly		
Study	Country of the Study	Sex	Alcohol	Tobacco	Level of Physical Activity	Muscle Mass	Sleep Quality	Age	Weight	Height	BMI	Type	Sex	Total	N	Cases	Control	N2	Cases	Control
Buchmann et al.	Berlin	Women	494	46	Moderate	DXA	PSQI	68			25.7	Cross-sectional	M	568	492	79	413	76	27	49
		Women	66	4	Moderate			69			31.2		W	628	508	64	444	120	13	107
		Men	424	59	Moderate			69			26.4		T	1196	1000	143	857	196	40	156
		Men	99	7	Moderate			69			30.2									
Chien et al.	Taiwan	Men			Regular	BIA	PSQI and Self-report	78.7	63.9	162.1	24.3	Cross-sectional	M	224	112	25	87	112	29	83
		Men			Regular			77.8	64.1	163.7	23.9		W	264	76	20	56	188	18	170
		Men			Regular			80	66.8	164.4	24.7		T	488	188	45	143	300	47	253
		Women			Regular			74.4	58.3	151.9	25.3									
		Women			Regular			74.5	57.9	153	24.8									
		Women			Regular			76.2	56.6	152.2	24.3									
Fu et al.	China	48.7% Men	No = 61.1%	No = 37.2%	Moderate	BIA	Self-report	68.24	70.24	163.38	25.9	Cohort study								
		40.5% Men	No = 62.2%	No = 38.9%	Moderate			66.3	67.96	163.91	25.3									
		37.9% Men	No = 59.5%	No = 33.7%	Moderate			67.38	67.16	163.39	25.1		T	920	468	52	416	452	43	409
		54% Men	No = 64.6%	No = 28.8%	Moderate			68.93	68.1	163.37	25.4									
Hu et al.	China	Men	57	62	Moderate	DXA	Self-report	70.8			23.6	Cross-Sectional Study	M	251	63	13	50	188	28	160
		Men	16	19	Moderate			72.6			18.7		W	356	63	14	49	293	57	236
		Women	16	1	Moderate			69.1			23.6		T	607	126	27	99	481	85	396
		Women	5	2	Moderate			72.3			20.3									
Ida et al.	Japan	Men	60%	72.1%		Self-report	PSQI	71.8			24.3	Cross-sectional study	M	189	105	14	91	84	22	62
		Women	17.2%	4,9%				72.8			23.9		W	129	71	11	60	58	24	34
													T	318	176	25	151	142	46	96
Kwon et al.	Korea	Men = 5819; Women = 8118	4.209	3.579	Regular	DXA	Self-report	44				Cross-sectional study	M							
		Men = 1339; Women = 872	635	797	Regular			45.2					W							
													T	16148	4938	819	4119	11210	1486	9724

M, men; W, women; T, total; DXA, densitometry; BIA, bioelectrical impedance analysis and PSQI, pittsburgh sleep quality index.

**Table 2:** The score obtained by the studies on the quality scale.

		Q1	Q2	Q3	Q4	Q5	Q6	M.S.	Qi
Buchmann et al. 2016	[19]	1	1	2	3	1	2	10	1
Chien et al. 2015	[36]	1	1	2	3	1	2	9	1
Fu et al. 2019	[37]	1	1	0	3	1	2	7	0.8
Hu et al. 2017	[38]	1	1	2	3	1	2	10	1
Ida et al. 2019	[34]	1	0	0	3	0	2	7	0.6
Kwon et al. 2017	[39]	1	1	2	3	1	2	10	1

TS, Total score; Q1, Were the target population and the observation period well defined?; Q2, Diagnostic criteria; Q3, Method of case ascertainment; Q4, Administration of measurement protocol; Q5, Catchment Area; Q6, Prevalence measure. M.S. mean score; Qi stands for a quality rank.

**Table 3:** Prevalence. Pooled results and CIs for three categories by transformation method and model.

Model	Sarcopenia and Self-Report or PSQI									Sarcopenia and Self-Report						
	Transf.	Category	Pooled	LCI	HCI	I^2^ (%)	Cochran’s Q	χ^2^ (*p*)	tau2/Q-Index	Pooled	LCI	HCI	I^2^	Cochran’s Q	χ^2^ (*p*)	tau2/Q-Index
Inverse Variance	None	SW	0.056	0.053	0.059	95.786	118.642	0.000		0.052	0.049	0.055	94.549	55.035	0.000	
		SP	0.088	0.084	0.091	95.786	118.642	0.000		0.090	0.086	0.094	94.549	55.035	0.000	
	Logit	SW	0.384	0.052	0.058	95.241	105.072	0.000		0.363	0.049	0.055	90.653	32.094	0.000	
		SP	0.616	0.084	0.092	95.241	105.072	0.000		0.637	0.087	0.095	90.653	32.094	0.000	
	Double arcsine	SW	0.388	0.052	0.059	95.847	120.389	0.000		0.363	0.049	0.055	93.072	43.300	0.000	
		SP	0.612	0.084	0.092	95.847	120.389	0.000		0.637	0.087	0.095	93.072	43.300	0.000	
Random effects	None	SW	0.073	0.044	0.102	95.786	118.642	0.000	0.001	0.060	0.030	0.091	94.549	55.035	0.000	0.001
		SP	0.090	0.061	0.119	95.786	118.642	0.000	0.001	0.093	0.062	0.124	94.549	55.035	0.000	0.001
	Logit	SW	0.460	0.046	0.103	95.241	105.072	0.000	0.264	0.399	0.041	0.083	90.653	32.094	0.000	0.126
		SP	0.540	0.055	0.119	95.241	105.072	0.000	0.264	0.601	0.062	0.122	90.653	32.094	0.000	0.126
	Double arcsine	SW	0.453	0.044	0.102	95.847	120.389	0.000	0.018	0.395	0.036	0.086	93.072	43.300	0.000	0.011
		SP	0.547	0.056	0.120	95.847	120.389	0.000	0.018	0.605	0.061	0.123	93.072	43.300	0.000	0.011
Quality effects	None	SW	0.056	-0.001	0.113	95.786	118.642	0.000	1.698	0.052	0.000	0.104	94.549	55.035	0.000	1.356
		SP	0.088	0.031	0.145	95.786	118.642	0.000	1.698	0.091	0.039	0.143	94.549	55.035	0.000	1.356
	Logit	SW	0.384	0.024	0.118	95.241	105.072	0.000	1.714	0.362	0.028	0.093	90.653	32.094	0.000	0.804
		SP	0.616	0.040	0.182	95.241	105.072	0.000	1.714	0.638	0.051	0.158	90.653	32.094	0.000	0.804
	Double arcsine	SW	0.379	0.011	0.112	95.847	120.389	0.000	1.583	0.353	0.015	0.096	93.072	43.300	0.000	1.013
		SP	0.621	0.030	0.155	95.847	120.389	0.000	1.583	0.647	0.042	0.148	93.072	43.300	0.000	1.013

Transf., transformation; HCI, higher CI; LCI, lower CI; SW, sleep well; SP, sleep poorly.
